# Ouabain Enhances Cell-Cell Adhesion Mediated by β_1_ Subunits of the Na^+^,K^+^-ATPase in CHO Fibroblasts

**DOI:** 10.3390/ijms20092111

**Published:** 2019-04-29

**Authors:** Claudia Andrea Vilchis-Nestor, María Luisa Roldán, Angelina Leonardi, Juan G. Navea, Teresita Padilla-Benavides, Liora Shoshani

**Affiliations:** 1 Department of Physiology Biophysics and Neurosciences, Center for Research and Advanced Studies, Cinvestav-Ipn, CDMX 07360, Mexico; cvilchis85@gmail.com (C.A.V.-N.); mroldan@fisio.cinvestav.mx (M.L.R.); 2 Department of Biochemistry and Molecular Pharmacology, University of Massachusetts Medical School, Worcester, MA 01605, USA; 3 Department of Chemistry, Skidmore College, 815 North Broadway, Saratoga Springs, NY 12866, USA; aleonar1@skidmore.edu (A.L.); jnavea@skidmore.edu (J.G.N.)

**Keywords:** Na^+^,K^+^-ATPase, β_1_ subunit, Ouabain, cSrc, pNaKtide, cell adhesion

## Abstract

Adhesion is a crucial characteristic of epithelial cells to form barriers to pathogens and toxic substances from the environment. Epithelial cells attach to each other using intercellular junctions on the lateral membrane, including tight and adherent junctions, as well as the Na^+^,K^+^-ATPase. Our group has shown that non-adherent chinese hamster ovary (CHO) cells transfected with the canine β_1_ subunit become adhesive, and those homotypic interactions amongst β_1_ subunits of the Na^+^,K^+^-ATPase occur between neighboring epithelial cells. Ouabain, a cardiotonic steroid, binds to the α subunit of the Na^+^,K^+^-ATPase, inhibits the pump activity and induces the detachment of epithelial cells when used at concentrations above 300 nM. At nanomolar non-inhibiting concentrations, ouabain affects the adhesive properties of epithelial cells by inducing the expression of cell adhesion molecules through the activation of signaling pathways associated with the α subunit. In this study, we investigated whether the adhesion between β_1_ subunits was also affected by ouabain. We used CHO fibroblasts stably expressing the β_1_ subunit of the Na^+^,K^+^-ATPase (CHO β_1_), and studied the effect of ouabain on cell adhesion. Aggregation assays showed that ouabain increased the adhesion between CHO β_1_ cells. Immunofluorescence and biotinylation assays showed that ouabain (50 nM) increases the expression of the β_1_ subunit of the Na^+^,K^+^-ATPase at the cell membrane. We also examined the effect of ouabain on the activation of signaling pathways in CHO β_1_ cells, and their subsequent effect on cell adhesion. We found that cSrc is activated by ouabain and, therefore, that it likely regulates the adhesive properties of CHO β_1_ cells. Collectively, our findings suggest that the β_1_ subunit adhesion is modulated by the expression levels of the Na^+^,K^+^-ATPase at the plasma membrane, which is regulated by ouabain.

## 1. Introduction

The Na^+^,K^+^-ATPase or sodium pump is an ubiquitous plasma membrane transporter that creates the ionic gradients that drive the net movement of glucose, amino acids, and ions across cellular membranes [1,2]. The Na^+^,K^+^-ATPase belongs to the P-type ATPase family, whose members are characterized by the transitory formation of a phosphorylated enzyme intermediate [3,4]. Structurally, the pump consists of three subunits: a catalytic α subunit, an accessory β subunit and a regulatory γ subunit. The catalytic α subunit consists of 10 transmembrane domains (TM), and exchanges 3 Na^+^ ions from the cytosol for 2 K^+^ ions from the extracellular milieu using the energy released from ATP hydrolysis [5]. The β subunit is constituted by a single TM domain, and a long glycosylated extracellular domain. Its functions are discussed in detail below. The γ subunit is a small, single span TM protein belonging to the FXYD family, which is differentially expressed in tissues and modulates the pump’s function [6,7]. In mammals, there are four α subunit isoforms, three β subunit isoforms and seven FXYD members [2,8].

The β subunit of the sodium pump has different functions that depend on the isoform expressed (β_1_, β_2_ or β_3_), and on the accompanying α subunit isoform (α_1_–α_4_) [9]. The main function of the β subunit is to act as a chaperone for the α subunit, by contributing to the assembly and delivery of the pump to the plasma membrane [10,11]. In addition, the β subunit undergoes conformational changes during the catalytic cycle [12]. Different β isoforms have been associated with different K^+^ affinities [13]. Furthermore, some studies suggest that the β_1_ subunit regulates cell polarity, cell motility, epithelial to mesenchymal transition, and oncogenic transformation [14,15,16]. In epithelia, the β_1_ isoform functions as a homophylic cell adhesion molecule [17,18,19]. Moreover, the β_2_ isoform is an adhesion molecule on glia (AMOG, [20]).

Emerging evidence has shown that the Na^+^,K^+^-ATPase may have other regulatory functions in addition to pumping ions across cell membranes. Ouabain and other related cardiotonic steroids are highly specific Na^+^,K^+^-ATPase ligands that bind to all catalytic α isoforms [21,22,23]. Studies from various laboratories have documented an important signaling function of the Na^+^,K^+^-ATPase [9,24]. In epithelia, the sodium pump also acts as a membrane receptor that transduces signals in response to ouabain and other related cardiotonic steroids. The binding of ouabain and cardiotonic steroids (at nM concentrations) to the sodium pump activates signaling pathways that resemble those triggered by hormone/receptor interactions, which, in turn, regulate gene expression, membrane trafficking, cell adhesion, proliferation, and cell death [25,26,27,28,29,30]. Interestingly, nanomolar concentrations of ouabain neither inhibit K^+^ pumping nor disturb the K^+^ balance of the cell [31]. Therefore, it was proposed that low ouabain concentrations bind and activate a non-pumping population of the Na^+^,K^+^-ATPase [32,33]. However, the nature of the effects that ouabain exerts at different concentrations in organisms is still controversial [34,35,36].

It was suggested that Ouabain is a hormone when Hamlyn and Mathews demonstrated the presence in plasma of a substance similar to ouabain in plants [22,37]. Thereafter, it was shown that endogenous ouabain is synthesized and secreted by the hypothalamus [38,39] and the adrenocortical gland [40,41,42]. The status of ouabain as a hormone was strengthened upon the discovery of increased concentrations in plasma during exercise, ingesting salty foods, and in pathological conditions such as arterial hypertension and myocardial infarction [43,44,45,46,47,48]. However, its physiological role remained unknown. Work from our group has shown that ouabain binding to the Na^+^,K^+^-ATPase modulates epithelial cell adhesion and communication [31,49,50].

Our laboratory has studied the role of the Na^+^,K^+^-ATPase β_1_ subunit in epithelia. We demonstrated that the β_1_ subunits of Na^+^,K^+^-ATPases on neighboring cells interact with each other in a species-specific manner [17,19]. Numerous studies have shown that the intercellular homotypic interaction between β_1_ subunits of the Na^+^,K^+^-ATPase are important for the stability of adherent junctions (AJ) and for the integrity of the tight junctions (TJ) [18,51]. Thus, β_1_-β_1_ interactions between epithelial cells are critical for the integrity of intercellular junctions. Since ouabain modulates different cell-attachment complexes, we wondered whether ouabain also regulates the β_1_-β_1_ mediated cell adhesion. In this work we used CHO fibroblasts, which lack the classical cell-cell adhesion complexes (TJs, AJ) and that stably express a canine β_1_ subunit in the plasma membrane. This model system targets the Na^+^,K^+^-ATPase efficiently to the plasma membrane contributing to the cell-cell contact [19]. To determine whether β_1_-β_1_ interactions are modulated by ouabain, we investigated the effect of a low dose (50 nM) of ouabain on the adhesion of CHO cells overexpressing the canine β_1_ subunit. This work shows that ouabain increases the amount of Na^+^,K^+^-ATPase at the cell membrane, resulting in increased cell adhesion properties that are mediated by β_1_-β_1_ interactions. This effect is facilitated by the ouabain-dependent activation of kinases such as cSrc and AKT, which enhance the adhesive properties of CHO β_1_ cells.

## 2. Results

### 2.1. Cell-Cell Adhesion of CHO Fibroblasts Expressing Canine β_1_ Subunit of Na^+^,K^+^-ATPase is Mediated by β_1_ Homotypic Interactions in-trans

**Figure 1:**
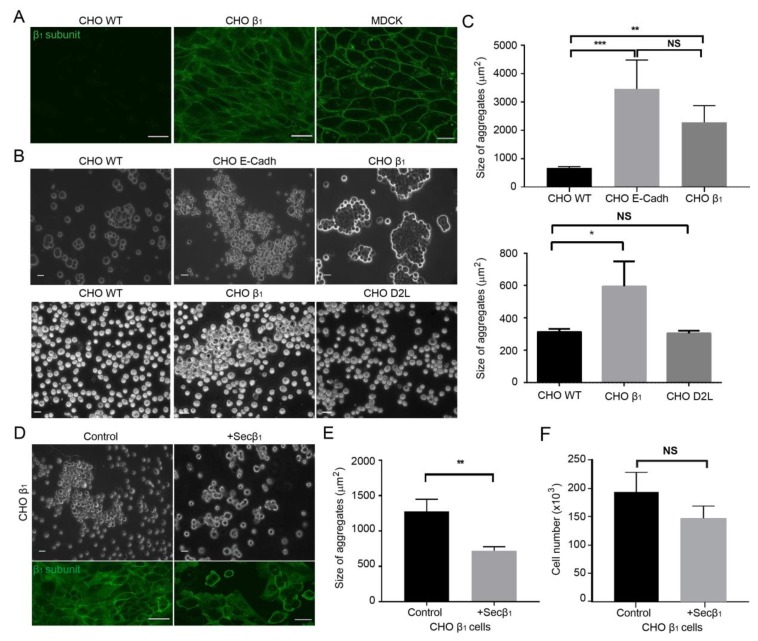
Cell-cell adhesion of CHO fibroblasts expressing the canine β_1_ subunit of Na^+^,K^+^-ATPase (CHO β_1_) is mediated by β_1_-β_1_ interactions. (**A**) Representative confocal microscopy images of CHO wild type, CHO β_1_ and MDCK cells immunostained against the canine β_1_ subunit. Scale bar = 30 μm. (**B**) Representative light microscopy images of CHO wild type, CHO β_1_, CHO E-Cadherin, and CHO D2L cells after the dispase aggregation assay. Scale bar = 20 μm. (**C**) Quantification of the size of the aggregates is depicted as the area of their horizontal projections. Values represent the mean from three independent biological replicates ± SE. Kruskal Wallis and Dunnet’s t-test for multiple comparisons were performed; NS, non-significant, * *p* < 0.05, ** *p* < 0.005, *** *p* < 0.0001. (**D**) Upper panels are representative phase-contrast micrographs of aggregation assays as in (**B**). Scale bar = 20 μm. Lower panels are representative confocal microscopy images of the canine β_1_ subunit in CHO β_1_ cells incubated for 24 h in the absence (left) or presence (right) of Secβ_1_. (**E**) Quantification of the mean size of cellular aggregates of untreated CHO β_1_ cells or cells treated with Secβ_1_. Student t-test of three independent biological experiments ± SD was performed; ** *p* < 0.005. (**F**) Proliferation assay of CHO β_1_ cells incubated for 24 h in the absence or presence of Secβ_1_. Student t-test of three independent biological experiments ± SD was performed; NS, non-significant.

Adherent CHO fibroblasts attach to the extracellular matrix and to their substrate, but establish weak cell-cell contacts, which are easily disrupted by gentle shaking or pipetting [19,52,53]. We have shown that CHO fibroblasts transfected with the canine β_1_ subunit of the Na^+^,K^+^-ATPase (CHO β_1_) form large cellular aggregates, due to an increase in cell-cell adhesion [19]. Moreover, we demonstrated that the epithelial β_1_ subunit of Na^+^,K^+^-ATPase is an adhesion molecule that mediates the interaction between sodium pumps on neighboring cells by establishing homotypic interactions [17]. Therefore, we hypothesized that the cell-cell adhesion observed in CHO β_1_ cells is mediated by β_1_-β_1_ interactions. To address this question, we investigated the subcellular localization of the canine β_1_ subunit and the cell-cell adhesion properties of CHO cells. Wild type CHO cells do not express the β_1_ subunit of the sodium pump (Figure 1A, left panel). Confocal microscopy analyses showed that CHO cells transfected with the plasmid encoding the canine β_1_ subunit express the protein in the plasma membrane, thereby showing a distribution resembling that observed in epithelial MDCK cells (Figure 1A, middle and right panels). Dispase assays showed that wild type CHO cells are unable to maintain cellular aggregates (Figure 1B, left panel). However, the cell-cell adhesion capability of CHO cells increased upon transfection of the canine β_1_ subunit similar to those of the CHO cells expressing the adhesion molecule E-cadherin, as these cells maintain larger cellular aggregates upon dispase disruption (CHO E-cadh; Figure 1B). On average, the aggregates formed by CHO β_1_ cells are significantly larger (3 fold) than control wild type CHO cells but similar in size to those observed in the CHO E-cadh cells (Figure 1C). Notably, CHO cells transfected with a plasmid encoding an irrelevant protein, the dopamine receptor 2 (CHO D2L, Figure 1 and Figure S1), failed to display adhesive properties similar to those of CHO β_1_ and CHO E-cadh cells (Figure 1B,C).

To confirm the hypothesis that the cell-cell adhesion observed in CHO β_1_ cells is due to β_1_-β_1_ interactions, we tested whether the soluble domain of the β_1_ subunit would impair the formation of cellular aggregates in this cell line. We took advantage of a truncated version of the canine β_1_ subunit that only expresses the soluble extracellular C-terminal domain (Secβ_1_) [17,54]. CHO β_1_ cells were allowed to interact with supernatants obtained from CHO Secβ_1_ cells containing this protein, and the formation of cellular aggregates was analyzed by light microscopy. Figure 1D shows that the presence of the soluble domain of the canine β_1_ subunit (Secβ_1_) reduced the size of the CHO β_1_ cellular aggregates. Statistical analyses confirmed that the aggregates formed by CHO β_1_ cells were significantly smaller (~50%) than those formed by control cells (Figure 1E). Interestingly, confocal microscopy and cell quantification analyses showed that CHO β_1_ cells pre-incubated for 24 h with Secβ_1_ supernatant presented a non-significant but consistent decrease in proliferation when compared to control cells (Figure 1D, lower panel, F). Remarkably, as can be observed in the IF images of Figure 1D (lower panel), contact naïve CHO β_1_ cells treated with Sec β_1_ unexpectedly express the β_1_ subunit at the plasma membrane and showed an intense and quantifiable fluorescence similar to the one observed in cell-cell contacts. These results confirmed that Na^+^,K^+^-ATPase- dependent cell-cell adhesion is at least partially due to an interaction between β_1_ subunits, and further showed that the cell culture model based on CHO β_1_ cells is suitable for studying β_1_-β_1_ interactions.

### 2.2. Ouabain Increases Cell-Cell Adhesion of CHO β_1_ Cells

**Figure 2:**
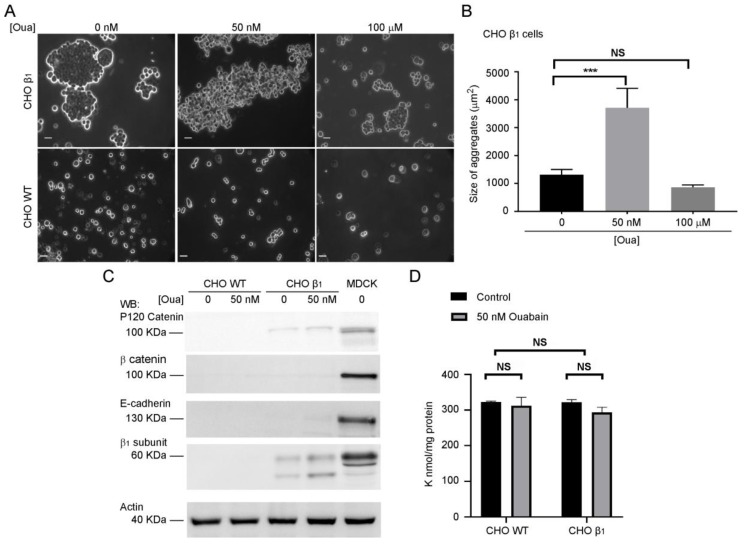
Low doses of ouabain increased cell-cell adhesion of CHO β_1_ cells. (**A**) Representative microscopy images of cell aggregation of CHO β_1_ cells (upper panels) and CHO WT cells (lower panels) treated with ouabain (50 nM, 100μM). Scale bar = 20 μm. (**B**) Quantification of the mean size of cellular aggregates of CHO β_1_ cells treated with 0, 50 nM and 100 μM ouabain based on three independent biological replicates. One way ANOVA and Dunn’s tests for multiple comparison were performed. Bars represent ± SE; NS, non-significant, *** *p* < 0.0001. (**C**) Representative Western blot analyses of the adhesion markers p120 catenin, β catenin, E-cadherin, and the β_1_ subunit of the sodium pump in CHO β_1_ and CHO WT cells, untreated or treated with 50 nM of ouabain. MDCK cells were used as positive control. Actin was used as loading control. (**D**) Whole cell content of K^+^ was determined by AAS from CHO β_1_ and CHO WT cells treated with 50 nM of ouabain for 24 h. The average of three independent biological replicates is depicted. One-way ANOVA and Dunn’s tests for multiple comparison were performed, bars represent ± SE, NS, non-significant.

Nanomolar concentrations of ouabain modulate cell-cell interactions [29,31]. Therefore, we hypothesized that ouabain may also control the cell-cell interactions that are mediated by the β_1_ subunits of the sodium pump. To test this hypothesis, we used the dispase adhesion assay, to further investigate the adhesive properties of CHO β_1_ cells in the absence or presence of ouabain. Figure 2 shows a specific and significant increase of the size of CHO β_1_ cell aggregates upon treatment with ouabain at a concentration of 50 nM (compare Figure 2A upper and lower panels). Importantly, the inhibitory ouabain concentration (100 μM) decreased the cell adhesion phenotype observed in CHO β_1_ cells to that of wild type cells. Therefore, we concluded that low doses of ouabain (50 nM) increase cell adhesion mediated by β_1_-β_1_ interactions of CHO β_1_ cells. Previous studies from our laboratory showed that the adhesive properties of CHO β_1_ cells do not involve the expression of adhesive proteins such as E-cadherin or β-catenin [19]. Consistent with this, we were unable to detect the expression of the adhesion markers β-catenin or E-cadherin in wild type and CHO β_1_ fibroblasts (Figure 2C). However, a small increase in the expression of p120 catenin was detected. Importantly, we utilized atomic absorbance spectroscopy (AAS) to evaluate the levels of Na^+^ and K^+^ in wild type and CHO β_1_ cells treated with 50 nM of ouabain. Figure 2D showed that the intracellular levels of K^+^ remained stable in untreated CHO wild type (CHO WT) and CHO β_1_ cells as well as in the fibroblasts treated with ouabain. The adhesive phenotype observed in the cells treated with a low dose of ouabain was specific to this cardiotonic steroid. Dispase assays and Western blot analyses showed that CHO β_1_ cells incubated with digoxin (50 and 100 nM) are unable to form cellular aggregates and that the induction of the β_1_ subunit does not occur with this cardiotonic steroid (Figure S2A,B). Furthermore, the intracellular levels of K^+^ in these cells also remained stable as shown by AAS analyses (Figure S2C). It is noteworthy that Na^+^ measurements by AAS were below the detection limit of this technique. All the samples tested had a Na^+^ concentration below 0.3 ppb in solution. Since no variations were detected between cells treated either with ouabain, or digoxin and non-treated cells, the data suggest that Na^+^ also remained stable in these cells.

### 2.3. The Interactions of β_1_-β_1_ Subunits are Stable in vitro Independently of Ouabain Treatment

**Figure 3:**
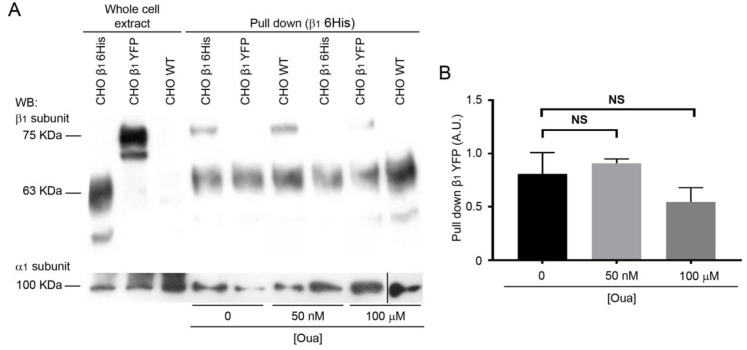
A direct interaction between ouabain and the α_1_ subunit of Na^+^,K^+^-ATPase is not sufficient for the observed adhesive phenotype. (**A**) Western blot analysis of β_1_ and α_1_ subunits of the Na^+^,K^+^-ATPase: cell lysate of CHO β_1_ 6His, CHO β_1_ YFP and CHO WT cells (three left lanes); Pull Down assay between CHO β_1_ 6His as a bait and CHO β_1_ YFP or CHO WT as a prey with 0 nM, 50 nM and 100 µM Ouabain (six right lanes). The 63 KDa band corresponds to the β_1_ 6His, and the higher-molecular-weight band corresponds to the β_1_ YFP (75 KDa), both recognized by the same anti-β_1_ subunit antibody. (**B**) Densitometric quantification of the results presented in A. In pulled down fractions, the density of the β_1_ YFP band was normalized with the β_1_ 6His band. Data represent the mean of three independent replicates. Kruskal Wallis and Dunn’s test for multiple comparisons were performed. Error bars, ± SE; NS, non-signigicant.

The effect of ouabain on cell-cell interactions mediated by β_1_ subunits could be due to the induction of a conformational change in β_1_ subunits by this cardiotonic steroid, resulting in a more adhesive molecule. Accordingly, ouabain binding to its receptor, the α subunit of the pump, should be sufficient for inducing the same effect on β_1_-β_1_ interactions in vitro. Therefore, we studied whether ouabain directly affects β_1_-β_1_ subunits interaction in a pull-down assay. In this case, we used the canine β_1_ tagged with a hexa-histidine repeat (CHO β_1_ 6His) immobilized on Ni^+^-NTA as the bait. The prey was obtained from total cellular extract of CHO β_1_ cells tagged with the yellow fluorescent protein (CHO β_1_ YFP). CHO WT cells were used as negative control. Cellular extracts (bait and prey) were allowed to interact in the absence or presence of ouabain, and the formation of the interacting β_1_ subunit complexes were analyzed by Western blot. Figure 3 shows that the immobilized CHO β_1_ 6His was able to interact with the recombinant β_1_ YFP obtained from cellular extracts. Importantly, the interaction in vitro was maintained, even in the presence of ouabain (Figure 3A). Statistical analyses demonstrated that ouabain treatment did not affect the amount of interacting proteins significantly (Figure 3B). All eluted fractions contained the α subunit, which means that the β_1_ 6His is assembled with the α subunit on the Ni^+^-NTA beads (Figure 3A). The data suggest that the effect of ouabain on cell-adhesion does not occur directly on β_1_ subunits, and that it is likely dependent on additional cellular components.

### 2.4. Ouabain Increases the Expression and Localization of the Sodium Pump at the Plasma Membrane

**Figure 4:**
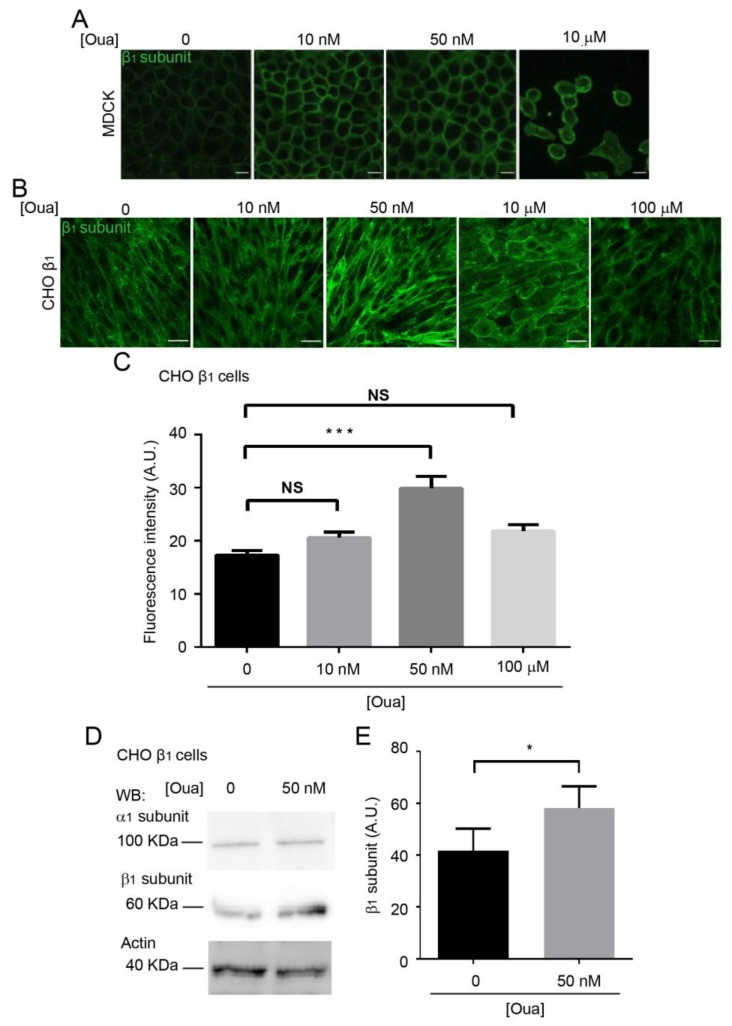
Ouabain increases the expression of the Na^+^,K^+^-ATPase at the plasma membrane of CHO fibroblasts transfected with the canine β_1_ subunit. (**A**) Representative confocal microscopy images of the canine β_1_ subunit in MDCK cells incubated with 10, 50 nM and 10 μM of Ouabain for 24 h, scale bar = 20 µm. (**B**) Representative confocal microscopy images of the canine β_1_ subunit in CHO β_1_ cells incubated for 24 h with 10, 50 nM, 10, and 100 μM of ouabain, scale bar = 20 µm. (**C**) Quantification of the fluorescence intensity observed at the plasma membrane of CHO β_1_ cells (10 cells per field of three independent biological replicates). One way ANOVA and Dunn´s test for multiple comparisons were performed. The data represent the mean ± SE; NS, non-significant, *** *p* < 0.0001. (**D**) Representative Western blot analysis of β_1_ and α_1_ subunits of the Na^+^,K^+^-ATPase of CHO β_1_ cells biotinylated after treatment with 50 nM ouabain for 24 h. Actin was used as a loading control for the input of the corresponding samples. (**E**) Densitometry quantification of biotinylated β_1_ subunit after treatment with ouabain. The data represent the mean of three independent biological replicates ± SD, Student t-test was performed; * *p* < 0.05.

It has been shown that treatment with cardiotonic steroids (ouabain and 21-benzylidene digoxin) increases the expression levels of the α subunit of sodium pumps in the porcine and canine kidney epithelium cell lines (LLC-PK1 and MDCK respectively) [55,56]. Therefore, we investigated whether the enhancing effect of ouabain on cell adhesion in CHO β_1_ cells was due to an increase in the expression of the sodium pump at the plasma membrane. As expected, treatment of MDCK cells with ouabain at nanomolar concentrations (10–50 nM) increased the expression of the β_1_ subunit at the plasma membrane (Figure 4A), which is in agreement with the observations of Rocha and coworkers (2014) [56]. On the other hand, treatment with ouabain at µM concentrations leads to the detachment of the MDCK cells (Figure 4A; [57]). Considering these phenotypes, we evaluated the effect of ouabain on the localization of the β_1_ subunit in the monolayer of CHO β_1_ cells. Confocal microscopy analyses showed that in our model system, the β_1_ subunit has a similar distribution in CHO cells to that in epithelial cells upon ouabain treatment (Figure 4B). In addition, the membrane localization of the sodium pump is disrupted at higher concentrations of ouabain (µM) (Figure 4B). Notably, the fluorescence intensity of the β_1_ subunit at the plasma membrane only increased significantly upon treatment with 50 nM ouabain (Figure 4C).

To confirm that nanomolar concentrations of ouabain increase the sodium pump expression at the plasma membrane, we used a surface biotinylation assay. Figure 4D shows a representative Western blot analysis of the β_1_ subunit located in the plasma membrane of CHO β_1_ cells treated with 50 nM ouabain. Statistical analyses of three independent biological experiments showed a small but significant increase in the membrane expression of the β_1_ subunit in CHO β_1_ cells (Figure 4D,E). Our results suggest that the observed increase in cell adhesion mediated by β_1_ subunits is a result of ouabain binding to the α subunit, which may lead to the activation of signaling pathways that promote the expression and delivery of the pump to the plasma membrane.

### 2.5. The β_1_-β_1_ Adhesion Enhanced by Ouabain Depends on the Activation of cSrc and AKT Signaling Pathway

**Figure 5:**
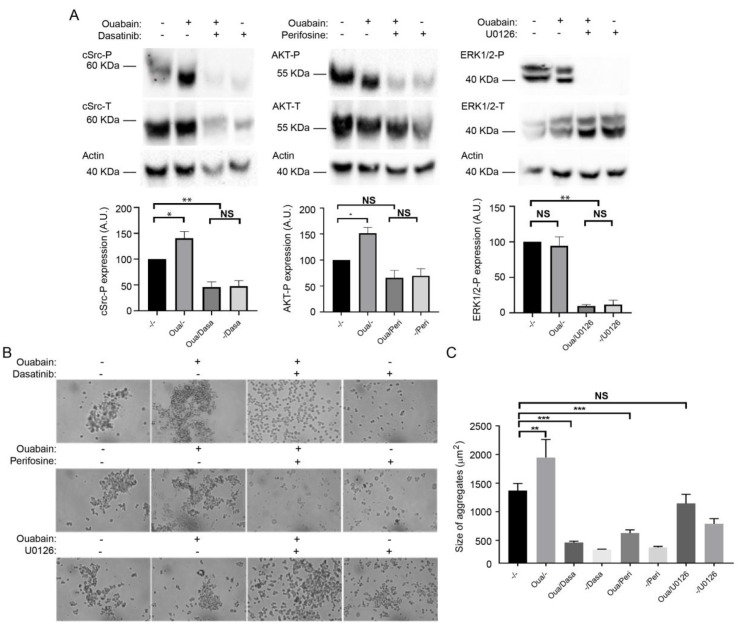
Src and AKT signaling pathways are involved in ouabain modulation of cell-cell adhesion of CHO β_1_ fibroblasts. (**A**) Representative Western blot analysis (upper panel) and quantification (lower panel) of cSrc-P, AKT-P, and ERK1/2-P of non treated (−) CHO β_1_ cells, and independent cultures treated with 50 nM ouabain (+), and in the presence (+) or absence (−) of the specific kinase inhibitor: cSrc (Desatinib, 100 nM); AKT (Perifosine, 10 μM); and EKR1/2 (U0126, 10 μM) (*n* = 3). The inhibitors were added 1 h prior to incubation with ouabain for 60 min. NS, non-significant, * *p* < 0.05, ** *p* < 0.005. (**B**) Representative images of dispase aggregation assay of untreated control (−/−) CHO β_1_ and cells treated with 50 nM ouabain and the specific inhibitors for cSrc, Desatinib; AKT, Perifosine; and EKR1/2, U0126. Image taken at a magnification 5×. (**C**) Quantification of the size of cellular aggregates. The data represent the mean of three independent biological replicates ± SE. Kruskal Wallis and Dunn´s test for multiple comparisons were performed. NS, non-significant, ** *p* < 0.005, *** *p* < 0.0001.

Previous studies demonstrated that binding of cardiotonic steroids such as ouabain to the sodium pump stimulates multiple kinase cascades [58]. Therefore, the effect of ouabain on the β_1_-β_1_ interaction could be explained by the potential activation of some components from the signaling machinery that are known to modulate cell junctions such as TJs, AJs and GAP junctions [31,59]. We first screened for signaling proteins of the tyrosine kinase family that were differentially activated in CHO β_1_ and CHO WT cells. Overall, CHO β_1_ cells had more active signaling proteins than wild type cells, indicating that the increased expression of Na^+^,K^+^-ATPase in CHO fibroblasts induces the activation of signaling cascades that usually have low activity (Figure S3). Then, we treated CHO β_1_ cells with 50 nM ouabain and identified various kinases that were altered when treating the ouabain cells (Figure S4). Of all the modified pathways detected, we focused on the potential mechanisms by which cSrc, AKT, and ERK1/2 contribute to the interaction between the β_1_ subunits. We selected these kinases, as they have been extensively reported by our group and others, to participate in cell adhesion and migration processes [49,60,61,62,63,64,65]. Western blot and quantitative band analyses from the three kinases selected showed that phosphorylated cSrc and AKT increased significantly upon treatment with 50 nM ouabain compared to control cells (Figure 5A). No significant phosphorylation effect of ERK1/2 was detected upon addition of ouabain (Figure 5A). The effect on kinase activation was also specific to ouabain, as treatment with digoxin failed to induce this effect (Figure S5). Then, we investigated whether the inhibition of those pathways would impair the effect of ouabain on cell adhesion mediated by β_1_ subunits. The selected phosphorylation inhibitors were Dasatinib for cSrc, Perifosine for AKT, and U01266 for ERK1/2. The incubation of the CHO β_1_ cells with all the inhibitors was effective and prevented the corresponding kinase phosphorylation in the presence or absence of ouabain (Figure 5A). Therefore, to test the effect of kinase inhibition on the ouabain-dependent adhesive properties of CHO β_1_ cells, we analyzed the size of the cellular aggregates. Representative light microscopy imaging showed that inhibition of cSrc and AKT impaired the enhanced ouabain-dependent formation of cellular aggregates (Figure 5B). No changes in the size of CHO β_1_ cellular aggregates in which ERK1/2 was inhibited were observed (Figure 5B). Statistical analyses showed significant differences in the size of CHO β_1_ cell aggregates treated with either perifosine or dasatinib; no significant differences were observed in CHO β_1_ cells treated with UO126 compared to control cells (Figure 5C). These results suggest that ouabain binding to the sodium pump stimulates cSrc kinase and AKT signaling pathways, which may be involved in the regulation of β_1_-β_1_ mediated cell adhesion.

### 2.6. pNaKtide Reduces Cell Adhesion Effect Exerted by Ouabain

**Figure 6:**
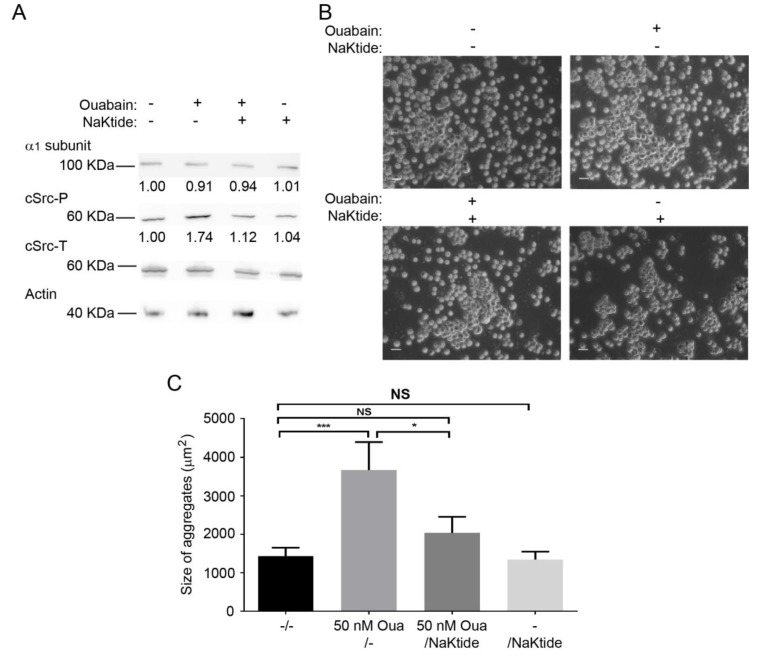
pNaKtide inhibits Ouabain-activated cSrc and ouabain enhanced β_1_-mediated cell adhesion in CHO β_1_ cells. (**A**) Representative Western blot of cSrc activation (cSrcP), total cSrc (cSrcT), and α_1_ subunits of CHO β_1_ cells treated with (+) or without (−) 50 nM ouabain and 1 µM pNaKtide for 24 h (*n* = 3). Actin was used as loading control. (**B**) Representative microscopy images of the dispase assay of CHOβ_1_ cells treated with (+) or without (−) 50 nM ouabain and 1 µM pNaKtide n for 24 h; scale bar = 20 μm. (**C**) Quantification of the size of cellular aggregates of CHO β_1_ control non-treated cells (−/−), and CHO β_1_ cells cultured with (+) or without (−) 50 nM ouabain and 1 µM pNaKtide. The data represent the mean of three independent biological replicates ± SE. Kruskal Wallis and Dunn´s test for multiple comparisons were performed. NS non-significant, * *p* < 0.05, *** *p* < 0.0001.

The Na^+^,K^+^-ATPase interacts with cSrc kinase and forms a complex that serves as a receptor for ouabain to stimulate various protein kinase cascades [33]. Specifically, ouabain binding to the Na^+^,K^+^-ATPase disrupts this interaction and results in the assembly and activation of different signaling pathways [66]. Thus far, our results have shown that the adhesive properties of β_1_ subunits require the cellular context, since no effect of ouabain was observed in vitro (Figure 3). Moreover, the increased phosphorylation of cSrc in CHO β_1_ cells treated with ouabain (Figure 5A), was reverted by the cSrc kinase inhibitor, and prevented formation of cellular aggregates (Figure 5B,C). Therefore, we hypothesized that the ouabain-dependent β_1_ subunit adhesive properties could be partially due to the activation of the Na^+^,K^+^-ATPase/cSrc complex. To test this hypothesis we took advantage of the pNaKtide, which is a peptide that antagonizes the effect of ouabain on cSrc [67], and assayed for cell-adhesion in the presence of ouabain with or without the pNaKtide. Figure 6A shows a representative Western blot of the ouabain-dependent increase in the phosphorylation of cSrc (~70% higher than control cells). In the presence of 1 μM of the pNaKtide, the effect on cSrc phosphorylation was abolished, and remained at similar levels to control cells. No changes were detected in the expression of the α subunit of the sodium pump. Finally, we analyzed the effect of the antagonist on the formation of ouabain-dependent aggregates of CHO β_1_ cells. Representative light microscopy images showed that the size of the antagonist-treated cell aggregates is smaller compared to the ouabain stimulated cells (Figure 6B). Statistical analyses showed that, in the presence of the pNaKtide, the size of ouabain-stimulated aggregates is similar to that of control cells (Figure 6C). These results support our hypothesis that the adhesion effect of β_1_ subunits stimulated by ouabain is partially regulated by cSrc activation.

## 3. Discussion

As all mammalian cells, CHO fibroblasts express the necessary amount of sodium pumps at their plasma membrane for maintaining a suitable ionic balance that maintains their viability. In our overexpression system, the assembled α_1_β_1_ dimer in CHO β_1_ cells is targeted to the plasma membrane and concentrated at cell-cell contacts, thereby leading to the adoption of an epithelial-like phenotype and to the maintenance of both cell-cell and cell-substrate adhesion [19]. This phenotype makes the CHO β_1_ overexpression system an ideal model to investigate cell adhesion. Our group has demonstrated previously that the β_1_ subunits of neighboring cells can interact directly in a species-specific manner by using the cellular system and biochemical tools described in this study [17]. We show that increasing the density of the β_1_ subunit of the Na^+^-pump in cell contacts of CHO fibroblasts is sufficient for displaying a cell-adhesive phenotype. These adhesive properties are mediated by β_1_ interactions, which are regulated by signaling cascades that ensure the establishment and maintenance of classical epithelial adhesion complexes [29,31,50].

Early work from the Nelson group on epithelial cell polarity showed that expression of E-cadherin in L-fibroblasts, which lack surface polarity, resulted in membrane domains with apical and basolateral identities [68]. Remarkably, the endogenous Na^+^-pumps in those experiments were recruited to cell-cell contacts. Our results show that CHO fibroblasts transfected with the β_1_ subunit, a Ca^2+^-independent adhesion molecule, or with E-cadherin, a Ca^2+^-dependent cell adhesion molecule, display similar aggregation properties. Moreover, overexpression of an irrelevant membrane protein, the Dopamine receptor D2L, did not induce cell-cell aggregation. Nevertheless, overexpression of β_1_ subunit in CHO cells does not induce the expression of classical adhesion markers such as E-cadherin and β catenin. Curiously, p120 catenin was detected by Western blot in CHO β_1_ cells, but not in WT cells. This observation could be related to the apparent “epithelialization” of the CHO β_1_ cells. To validate that cell-cell adhesion of CHO β_1_ cells was in fact due to β_1_-β_1_ interactions, cellular aggregation was challenged with Secβ_1_, the soluble extra-cellular domain of the canine β_1_ subunit. As expected, this truncated version of the β_1_ prevented the cell-cell adhesion properties observed in CHO β_1_ cells. These results strongly suggest that Secβ_1_ competes for cell-cell adhesion mediated by β_1_ subunits on neighboring cells and that the effect of ouabain on cell-cell adhesion is indeed through β_1_-β_1_ interactions. Remarkably, Secβ_1_ blocks cell-cell aggregation between CHO β_1_ cells only when it is added shortly after seeding and before the formation of a confluent monolayer. This observation indicates that Secβ_1_ is not able to compete with formed β_1_-β_1_ interactions but can interact with non-occupied β_1_ subunit and to prevent cell-cell adhesion mediated by β_1_ subunits. Moreover, CHO β_1_ cells pre-incubated with Secβ_1_ do not reach confluence, but rather form small patches or are observed as single cells. We wondered whether this was due to a proliferation defect. Our results show that the Secβ_1_ treated cells indeed tend to proliferate less than the untreated ones. Usually, contact naïve epithelial cells do not express the Na^+^-pump at the plasma membrane [69]. Notably, MDCK cells co-cultured with CHO WT cells do not express the Na^+^,K^+^-ATPase at the heterotypic contacting membrane [19]. Nevertheless, some of the CHO β_1_ single cells showed a highly fluorescent signal at the plasma membrane, which corresponded to the β_1_ subunit. Therefore, it is plausible that these contact naïve CHO β_1_ cells are actually surrounded by soluble Secβ_1_ molecules associated with the membrane-bound β_1_ subunit, thereby mimicking cell-cell contacts. As such, we hypothesize that the apparent proliferation defect is due to contact inhibition induced by Sec β_1_. These results lead to a new paradigm of the sodium pump- dependent modulation of cell proliferation. Altogether, these results confirm that cell-cell adhesion of CHO fibroblasts that overexpress Na^+^,K^+^-ATPase is mainly based on β_1_-β_1_ interactions between neighboring cells.

Ouabain binding to the α subunit of the epithelial Na^+^,K^+^-ATPase has a dual effect on the pump, whereby high concentrations of ouabain (>300 nM) trigger pump inhibition and cell detachment [49,57]. Low concentrations of ouabain increase sodium pump activity [70,71], and stimulate cellular signaling pathways [30]. In epithelial MDCK cells, ouabain concentrations between 10 and 100 nM are known to modulate cell contacts, such as TJs, AJs and GAP junctions [29,31,49,50]. We analyzed the effect of ouabain on the effect of β_1_-β_1_ mediated cell adhesion. Our results show that CHO β_1_ cells incubated with low concentrations of ouabain (50 nM) did not have altered K^+^ concentrations. However, this low dose allows the formation of bigger aggregates than those observed in non-treated cells. On the other hand, incubation with high concentration of ouabain (100 µM) partially prevented the cell-cell adhesion effect observed in CHO β_1_ cells. Since these cells remain partially adhesive, we cannot overrule the possibility of an effect of ouabain that is independent of the β_1_ subunit although we could not detect classical cell-adhesion markers such as E-cadherin and β-catenin in control or ouabain treated CHO β_1_ cells. Therefore, our results strongly suggest that low concentrations of ouabain induce an increase in β_1_-β_1_ interaction in CHO β_1_ cells.

We then asked whether ouabain had a direct effect on the β_1_-β_1_ interaction. Ouabain binding to the α subunit could induce a localized conformational change that would make the β_1_ subunit more adhesive which could be considered a direct effect. Ouabain binding produces conformational rearrangements in the α subunit of the sodium pump [72]. Furthermore, fluorometry assays identified three positions on the β_1_ subunit, that indicate that α and β subunits move toward each other during conformational transition and produce a conformational rearrangement [12,73]. To further assess this process, our in vitro experiments showed that although the α subunit is present during the immobilization on Ni-NTA and pull down assays, ouabain treatment does not modify β_1_-β_1_ interaction. As such, our results suggest that ouabain does not modify the adhesion between β_1_ subunits directly and that it requires additional cellular components to fulfill this function. In this regard, binding of ouabain to the α subunit could activate signaling cascades that would up-regulate the amount of Na^+^,K^+^-ATPase exposed to the extracellular space and thus indirectly increase cell-cell adhesion mediated by β_1_ subunits. It was established that epithelial cells express at least two different populations of the Na^+^,K^+^-ATPases: A pumping population and a signaling (non-pumping) population [25,66,74,75], which could be the case for transfected CHO β_1_ fibroblasts. Overexpression of the β_1_ subunit up-regulates the expression of the α subunit [32,76]. Both are assembled in the endoplasmic reticulum and targeted to the plasma membrane [77]. Consistent with this idea, confocal microscopy and surface biotinylation analyses performed in this study showed that ouabain stimulates the expression and delivery of the pump to the plasma membrane. Apparently, the signaling population is increased because we observed that, even without ouabain, the basal expression of cSrc, AKT, and ERK1/2 is increased in comparison to non-transfected CHO cells. Studies are now directed to understand the participation of the β_1_ subunit in the activation of additional signaling cascades in a ouabain-independent fashion (shown in Figure S3), and are beyond the scope of this manuscript. Moreover, nanomolar concentrations of ouabain stimulated the activation of cSrc; furthermore, the pNaKtide, which only binds to cSrc associated with Na^+^,K^+^-ATPase, successfully reverted the effect of ouabain on cSrc activation and cell-cell adhesion. Importantly, a fundamental difference between digoxin and ouabain is the apparent failure of digoxin to activate Src kinase [78]. This feature may explain the observed lack of an adhesive effect of digoxin. In addition, AKT seems to regulate the ouabain-dependent cell-cell adhesion observed in CHO β_1_ fibroblasts, as the specific inhibitor of AKT abolished the β_1_-mediated cell-cell adhesion. This is not surprising, as it has been shown that AKT trans-activates IP3K, which forms part of the signaling complex in association with Na^+^,K^+^-ATPase [28,79]. On the other hand, the inhibition of ERK signaling cascade had a minor effect on the ouabain-dependent cellular aggregation. This might be partially explained by the fact that ERK1/2 is not the only signaling effector activated by cSrc [80,81]. Moreover, our initial screening revealed various signaling proteins that were not further analyzed and that could be involved, such as TrkB, EphB4 and PDGFR. Future studies will focus on understanding the role of these signaling molecules in the β_1_-β_1_ adhesion phenotype.

All together, our results suggest that overexpression of the β_1_ subunit in CHO cells increases the population of Na^+^,K^+^-ATPases at the plasma membrane. This pool of pumps is most probably involved in mediating β_1_-β_1_ interactions between neighboring cells. Whether these proteins have an active role in ion transport remains to be elucidated, but considering the data obtained by AAS, it is unlikely that the role of these pumps is limited to ion transport. A plausible model could involve the activation of cSrc and AKT upon stimulation of CHO β_1_ cells with nanomolar concentrations of ouabain. These signaling cascades would result in the overexpression of adherent pumps targeted to cell contacts at the plasma membrane. Nonetheless, an open question that would have to be addressed in the future is if CHO β_1_ cells form signalosomes as epithelial cells do, and if β_1_-β_1_ interactions occur between specific pools (pumping and non-pumping pool). This study highlights the importance of the β_1_ subunit of the Na^+^,K^+^-ATPase and strengthens the new paradigm that this subunit of the sodium pump is a novel drug target to prevent cancer metastasis, where the deregulation of cell adhesion is a major detrimental pathological effect.

## 4. Materials and Methods

### 4.1. Cell Culture

Starter Cell lines, MDCK (Madin-Darby Canine Kidney, CCL-34) and CHO-K1 (Chinese hamster ovary) were obtained from the American Type Culture Collection. CHO-K1 cells were cultured in a mixture of F12/DMEM (1132-082, Invitrogen, Carlsbad, CA, USA), supplemented with 10% fetal calf serum (FCS) (200-6170, Invitrogen), 100 U/mL penicillin, and 100 μg/mL streptomycin (600-5145, Invitrogen) and MDCK cells were cultured in DMEM (430-1600, Invitrogen) supplemented with 10% FCS (200-6170, Invitrogen), 100 U/mL penicillin, and 100 μg/mL streptomycin (600-5145, Invitrogen). Cells were grown at 36.5 °C a 5% CO2 atmosphere, 90% humidity (Forma Scientific CO2 incubator, Steri-Cult 200).

For all experiments, the cells were depleted of serum 24 h before adding different concentrations of ouabain (3125, Sigma-Aldrich, St. Louis, MO, USA), or digoxin (458350, Tocris Bioscience, Bristol, UK) as indicated in the figures (10 nM, 50 nM, 100 nM 10 µM or 100 µM).

CHO-K1 cells were transfected with pCIN4-β_1_ (CHO β_1_), pIRESneo-6His (CHO β_1_ 6His), and the canine pEYFP-β_1_ (CHO β_1_ YFP) plasmids as previously described [17,19,51]. CHO-K1 cells transfected with EcDendra [82] were a kind gift of Dr. P. Nava (Cinvestav, Mexico). CHO-K1 cells expressing the dopamine receptor 2 (CHO D2L, pcDNA3.1 D2L) were a kind gift of Dr. J. A. Arias-Montaño (Cinvestav, Mexico) The construct for secreted ecto-domain of the canine β_1_ subunit (Secβ_1_) was a kind gift of Dr. D. M. Fambrough (Johns Hopkins University, USA), and was generated as previously reported [83]. Secβ_1_ was transfected in CHO cells as described by Padilla-Benavides, et al., 2010 [17]. CHO Secβ_1_ cells were cultured in serum free media for 4 days before collecting the conditioned medium containing the ecto-domain of β_1_ subunit [17,54]. Stable clones were selected and maintained with 0.2 mg/mL G418 (11811031, Thermo Fischer Scientific, Waltham, MA, USA ) in F12/DMEM mixture.

For experiments including Secβ_1_ the CHO β_1_ cells were seeded in 24 wells plates and after 90 min the monolayer was washed three times with PBS and a serum free medium with or without Secβ_1_ was added. The next day the medium was replaced with a medium containing ouabain and Secβ_1_.

The kinase inhibitors used were against cSrc (Dasatinib 100 nM), AKT (Perifosine 10 μM), ERK1/2 (U0126, 10 μM) (0952, 14,240 and 9903, respectively, Cell Signaling Technologies, Danvers, MA, USA) and pNaKtide (1 μM) was a kind gift of Dr. Z. Xie (Marshall Institute for Interdisciplinary Research, USA). Inhibitors were added 1 h before ouabain treatment.

### 4.2. Antibodies

The primary antibodies used were: mouse anti-α_1_-subunit of the Na^+^,K^+^-ATPase (7671, Abcam, Cambridge, UK). Mouse anti-β_1_-subunit of the Na^+^,K^+^-ATPase (kind gift of Dr. M. Caplan, Yale University, USA). Rabbit anti-phospho-Akt (Ser473, D9E), rabbit anti-phospho-p44/42 MAPK (Erk1/2, Thr202/Tyr204, D13.14.4E); rabbit anti-phospho-Src Family (Tyr416, D49G4); rabbit anti-Akt (pan, C67E7); rabbit anti-p44/42 MAPK (Erk1/2, 137F5), and rabbit anti-Src (36D10) (4060, 4370, 6943, 4691, 4695, and 2109, respectively, Cell Signaling Technologies) The following rabbit antibodies were obtained from Abclonal (Woburn, MA, USA): anti-Src total (A0324), anti-p120 catenin (A1641), anti-β catenin (A11343), anti-E cadherin (A3044), anti-phospho-Src-Y416 (AP0480), anti-ERK1/ERK2 total (A16686), anti-phospho-ERK1-T202/Y204 and ERK2-T185/Y187 (AP0472).

The secondary antibodies used were: goat anti-rabbit IgG, and goat anti-mouse IgG coupled to HRP (65-6120 and 62-6520, respectively, Thermo Fisher Scientific). Goat anti-mouse IgG Alexa 488-conjugated (A11094, Molecular Probes, Eugene, OR, USA).

### 4.3. Immunofluorescence and Confocal Microscopy Analyses

CHO and MDCK cells grown on coverslips at the indicated conditions in the figures, were immunostained as previously described [19]. Briefly, the cells were washed with PBS, fixed and permeabilized with ice-cold methanol for 5 min. After washing with PBS, the cells were blocked with 3% bovine serum albumin for 1 h, followed by 1 h incubation with a mouse primary antibody against the β_1_ subunit of the Na^+^,K^+^-ATPase (1:50 dilution) at room temperature. Cells were washed 10 times quickly with PBS, and incubated with a goat anti-mouse Alexa 488 secondary antibody (1:500 dilution) for 30 min at room temperature. After washing, the cells were mounted on glass slides with FluoroGuard antifade reagent (170–3140, BioRad, Hercules, CA, USA). Samples were imaged using a confocal microscope (Leica TCS SP8, Leica, Wetzlar, Germany), and visualized using the Leica Lite software. The relative fluorescence intensity was quantified using IMAGEJ Fiji 1.0 software [84].

### 4.4. Western Blot Analysis

Monolayers of CHO cells grown on multi-well plates were washed 3 times with ice-cold PBS and incubated in RIPA buffer (24948A, Santa Cruz Biotechnologies, Dallas, TX, USA) with protease inhibitors (2498, Santa Cruz) for 30 min under continuous and vigorous shaking. After that the cells were scraped and collected into a 1.5 mL microcentrifuge tube. The cell extract was homogenized with insulin syringe and centrifuged at 17,700× *g* for 10 min. The supernatant was recovered, and protein concentration was quantified by BCA as indicated by the manufacturer (23225, Pierce Chemical Co. Dallas, TX, USA). Thirty μg of protein were separated on 10% SDS-PAGE gels, electro-transferred onto a PVDF membrane (RPN 303F, Hybond-P Amersham Biosciences, Little Chalfont, UK), and detected using the indicated primary (anti-α_1_-subunit of the Na^+^,K^+^-ATPase (1:2000), mouse anti-β_1_-subunit of the Na^+^,K^+^-ATPase (1:500). The rabbit anti-phospho-Akt, anti-phospho-p44/42 MAPK, anti-phospho-Src, anti-Akt, anti-p44/42 MAPK, anti-Src, anti-E cadherin, anti-β catenin and anti-p120 catenin were used at a 1:2000 dilution. The appropriate secondary antibodies (goat anti-rabbit IgG, and goat anti-mouse IgG coupled to HRP and goat anti-mouse IgG Alexa 488-conjugated were used at a 1:10,000 dilution. Membranes were developed with ECL PLUS (RPN2132, Amersham Biosciences). Immunoblots were imaged by FUSIONFX (Vilber, Marne-la-Vallée, France) and ChemiDocXRS (BioRad), and quantified by densitometry using the IMAGEJ, Fiji 1.0 software [84].

### 4.5. Cell Surface Biotinylation

Confluent monolayers of CHO β_1_ cells were depleted of serum for 24 h and then incubated with or without 50 nM ouabain for 24 h. Then, the monolayers were washed 3 times with PBS, and incubated with 1 mg/mL of EZ-Link Sulfo-NHS-SS-Biotin (21331, Thermo Fischer Scientific) for 30 min. Subsequently the monolayers were washed 3 times with PBS containing 100 mM glycine to quench the excess of Biotin, followed by a final wash with PBS containing 1% Triton X-100 and protease inhibitors. After 30 min, the cells were scraped, and the cell lysate collected into a 1.5 mL microcentrifuge tubes. The extract was homogenized by passing it 10 times through insulin syringe and centrifuged for 10 min at 9000× *g* at 4 °C. The supernatant was recovered, and the protein content was measured using the BCA protein assay method. The biotinylated extracts were incubated overnight at 4 °C with 100 μL of streptavidin-agarose suspension (S1638, Sigma-Aldrich). The next day, bead-adherent complexes were washed 5 times with PBS, and finally the proteins were eluted in 2× Laemmli buffer and boiling for 5 min.

### 4.6. Cell Adhesion Assay (Dispase Assay)

Confluent Monolayers of CHO β_1_ cells seeded on 24 well plates were depleted of serum for 24 h, and then incubated with or without ouabain (50 nM) for 24 h. Then the monolayers were washed with ice-cold PBS, and detached from the plates by incubation with PBS without Ca^2+^ supplemented with 0.6 U/mL of Dispase I (D4818, Sigma-Aldrich) for 35 min at 37 °C. Subsequently the Dispase solution was carefully removed using a 200 µL pipette tip, and replaced by 100 μL of PBS. The cells were then mechanically stressed by pipetting up and down 5 times using a 200 µL pipette. The resulting aggregates were visualized by light microscopy using the 10 × and 20 × objectives (Axiovert 200M Fluorescence/Live Cell Imaging, Carl Zeiss, Oberkochen, Germany). Three independent biological replicates were imaged using the AxioVision 4.8 software. The number of aggregates was counted using the Fiji 1.0 software cell counter (aggregates < 200 μm^2^ were excluded from the quantification) [84].

### 4.7. Pull Down Assay (PD)

Total extract of CHO cells expressing the β_1_ 6His construct were immobilized with nickel-nitrilotriacetic acid beads (Ni^+^-NTA, His Trap FF column; GE Healthcare, Chicago, IL, USA) equilibrated with 10 mL of RIPA buffer containing protease inhibitors. Total protein extracts (500 µg) were loaded into the resin, and allowed to interact for 3 h at 4 °C with gentle shaking. Then the unbound protein was washed as indicated by the manufacturer, and the total extract of CHO β_1_ YFP cells incubated in the presence or absence of ouabain (50 nM and 100 µM) were loaded as a prey. Samples were incubated overnight at 4 °C, and washed with PBS supplemented with 10 and 20 mM imidazole. Interacting proteins were eluted in PBS containing 500 mM imidazole and were analyzed by Western blot.

### 4.8. Whole Cell K^+^ and Na^+^ Determinations by Atomic Absorbance Spectroscopy

The analysis of both potassium and sodium concentrations from cells obtained from 15 cm^2^ plates was carried out by at least triplicate measurements using atomic absorption spectroscopy (AAS) equipped with a graphite furnace (GF-AAS) (AAnalyst 800, PerkinElmer, Waltham, MA, USA) with hollow cathode lamps as the radiation source [85,86,87]. In order to reach the lowest possible limit of detection and eliminate possible contamination, the graphite tubes used were cleaned via high-temperature gas-phase procedures (UltraClean THGA Graphite Tubes, PerkinElmer). This technique allows trace elemental analysis at a relative low cost in low volume samples, were dilution can be limited by the low initial concentration of analyte in the sample. A known mass of sample was acid digested in concentrated HNO_3_, using a single-stage digestion method [86,87,88,89]. To avoid contamination in the trace elemental analysis, all reagents used for sample treatment and calibration standard preparation were analytical grade and, when appropriate, diluted in 18 MΩ purified water. All analytical glassware was acid washed overnight in 10% (*v*/*v*) HCl and rinsed with 18 MΩ purified water before used.

Sodium and potassium standard solutions (1000 mg/L from Sigma-Aldrich) were diluted to obtain appropriate standards and determine the limits of detection and dynamic range of the method. The limit of detection (LOD) for K and Na, calculated as 3σ, was 0.1 ppb and 0.3 ppb, respectively. The dynamic range for K and Na spam from their LOD to a maximum concentration of 150 ppb and 200 ppb, respectively. Sodium and potassium content on each sample was normalized to the initial protein concentration of cells.

### 4.9. Statistical Analysis

GraphPad Prism version 7.00 software (San Diego, CA, USA) was used for all statistical analyses. The data are presented, as the mean ± SEM or SD as indicated in the figures. Statistical significance was determined using ANOVA one way and Kruskal Wallis test, and *t*-test for two conditions. *p* ≤ 0.05 was considered significant.

## 5. Conclusions

Research has shown that the homotypic interactions between β_1_ subunits of the Na^+^,K^+^-ATPase contributes to the stability and integrity of AJs and TJs in epithelial cells. In addition, the cardiotonic steroid ouabain further modulates various cell-attachment complexes. The work presented here demosntrated that ouabain also regulates cellular adhesion mediated by β_1_-β_1_ subunits. We showed that the presence of β_1_ subunits of the Na^+^,K^+^-ATPase at the cell surface promotes cellular contacts, and these interactions were further enhaced by low doses of ouabain. The activation of ouabain-dependent kinases such as cSrc and AKT, are responsible for the increased adhesive properties observed in CHO β_1_ cells, which ultimately contribute to the establishment of a broader adhesive phenotype in cells.

## Supplementary Materials

Supplementary Materials can be found at https://www.mdpi.com/1422-0067/20/9/2111/s1.
