# Supplementary material for: Ouabain Enhances Cell-Cell Adhesion Mediated by β1 Subunits of the Na+,K+-ATPase in CHO Fibroblasts
Source: Int J Mol Sci. 2019 Apr 29;20(9):2111. doi: 10.3390/ijms20092111 (PMC6539428; doi:10.3390/ijms20092111)
Supplement: Supplementary file 1 [file ijms-20-02111-s001.pdf]

Ouabain Enhances Cell-Cell Adhesion Mediated by β1 Subunits of the Na^+,K^+-ATPase in CHO Fibroblasts

Claudia Andrea Vilchis-Nestor [1,2], María Luisa Roldán [1], Angelina Leonardi [3], Juan G. Navea [3], Teresita Padilla-Benavides [2,*] and Liora Shoshani [1,*]

1 Department of Physiology Biophysics and Neurosciences, Center for Research and Advanced Studies, Cinvestav-Ipn, CDMX, 07360, Mexico; cvilchis85@gmail.com (C.A.V.-N.); mroldan@fisio.cinvestav.mx (M.L.R.)
2 Department of Biochemistry and Molecular Pharmacology, University of Massachusetts Medical School. Worcester, MA 01605, USA
3 Department of Chemistry, Skidmore College, 815 North Broadway, Saratoga Springs, NY 12866, USA; aleonar1@skidmore.edu (A.L.); jnavea@skidmore.edu (J.G.N.)
* Correspondence: terepadillabenavides@gmail.com (T.P.-B.); shoshani@fisio.cinvestav.mx (L.S.); Tel.: +1-508-856-5204 (T.P.-B.); +52-55-57-47-3360 (L.S.)

SUPPLEMENTAL INFORMATION

FIGURE S1

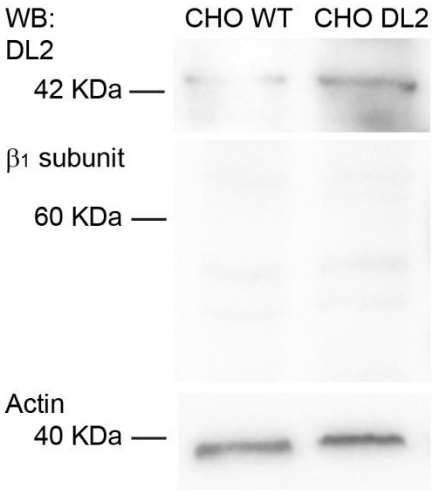

Figure S1. Expression of DL2 protein. Representative Western blot of CHO WT, and CHO DL2 fibroblasts expressing the Dopamine receptor 2. The β_1 subunit and actin were used as controls.

Figure S2

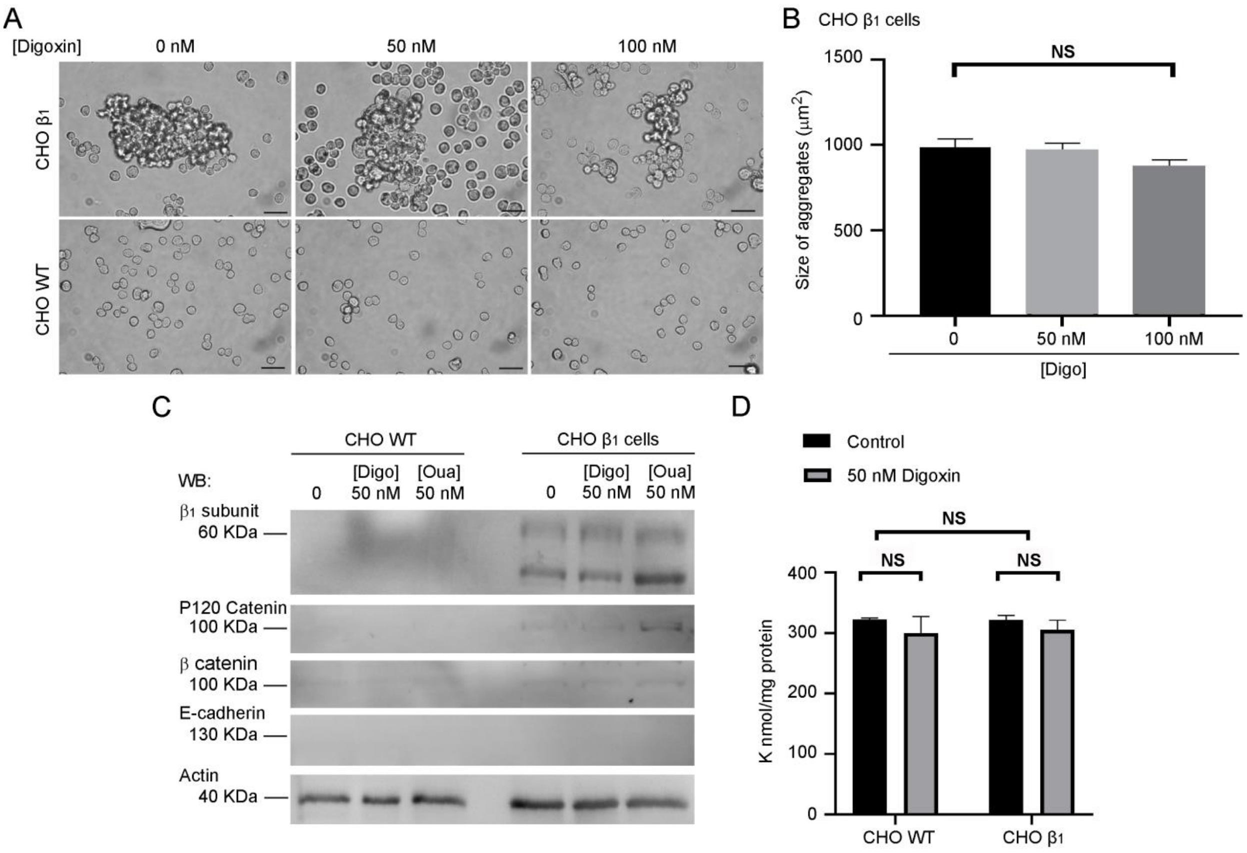

Figure S2. The effect of digoxin on cell-cell adhesion in CHO β1 fibroblasts is specific to this cardiotonic steroid. (A) Representative light microscopy images of CHO WT and CHO β1 cells treated with digoxin, after the dispase aggregation assay. Scale bar = 40 μm. (B) Quantification of the measured size of the aggregates is depicted as the area of their horizontal projections. Values represent the mean from three independent biological replicates ± SE. Statistical analysis was Kruskal Wallis and Dunnet's t-test for multiple comparisons. NS, non-significant. (C) Representative Western blot of CHO WT and CHO β1 cells treated or not with 50 nM digoxin or ouabain. The expression of adhesion molecules and kinases is depicted. Actin was used as loading control. Whole cell content of K^+ was determined by AAS from CHO β1 and CHO WT cells treated or not with 50 nM of ouabain for 24 h. The average of three independent biological replicates is depicted. Statistical analysis was ANOVA one way and Dunn's tests for multiple comparisons, bars represent ± SE. NS, non-significant.

Figure S3

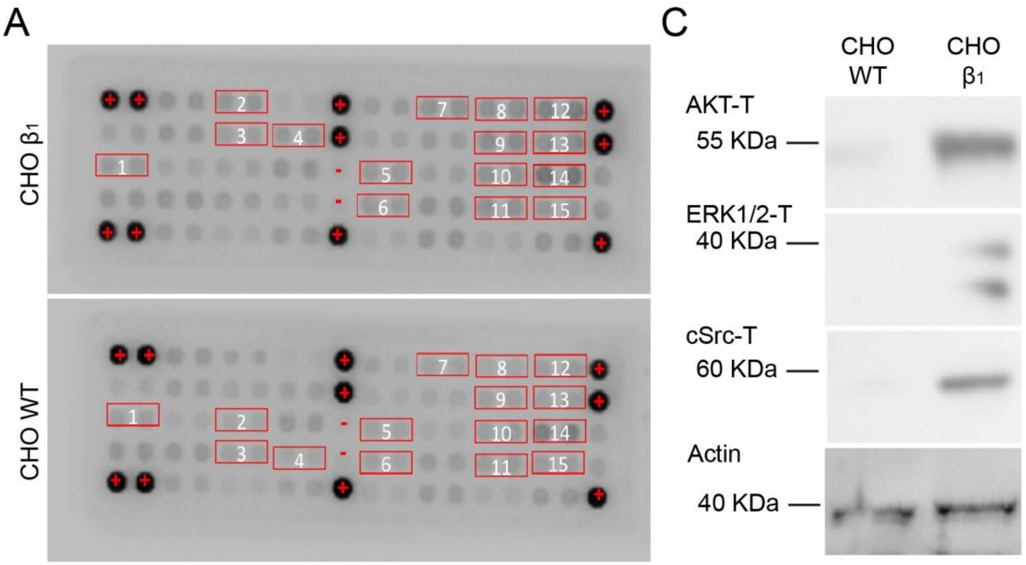

B

	Target	Phosphorilation site	Family
1	HER2/ErB2	Pan-Tyr	EGFR
2	TrkA/NTRK1	Pan-Tyr	NGFR
3	TrkB/NTRK2	Pan-Tyr	NGFR
4	PDGFR	Pan-Tyr	PDGFR
5	EphA3	Pan-Tyr	EphR
6	EphB1	Pan-Tyr	EphR
7	EphB4	Pan-Tyr	EPhR
8	AKT/PKB/Rac	Thr 308	Akt
9	AKT/PKB/Rac	Ser 473	Akt
10	p44742 MAPK(ERK1/2)	Thr 202/tyr 204	MAPK
11	S6 Ribosomal protein	Ser 335/236	RSK
12	IRS-1	Pan-Tyr	IRS
13	Zap-70	Pan-Tyr	Zap-70
14	Src	Pan-Tyr	Src
15	Lck	Pan-Tyr	Src

Figure S3. Phosphorylation state of major Receptor Tyrosine Kinases (RTKs) in CHO WT, and CHO β1 cells. (A) The RTK signaling antibody array performed with total extracts of CHO WT and CHO β1 cells incubated for 24 h without serum. Each point represents one RTK (PathScan RTK Signaling Antibody Array Kit). (B) List of proteins that showed an apparent increase their phosphorylated state in CHO β1 cells compared to CHO WT cells. (C) Representative Western blot (n=2) showing the expression of AKT-T, ERK 1/2-T and Src-T in CHO WT and CHO β1 cells with specific antibodies.

FIGURE S4

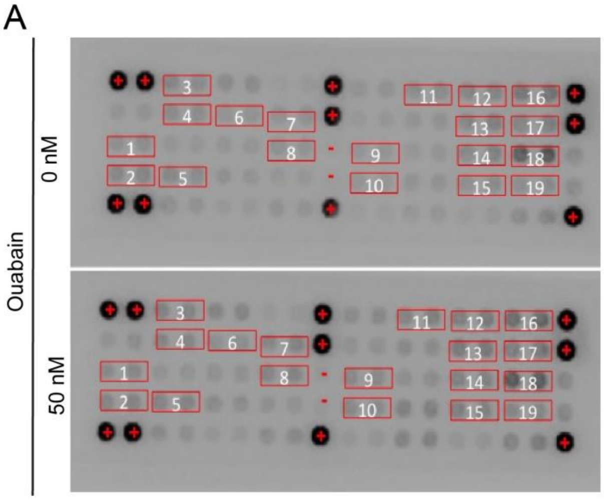

B

	Target x	Phosphorilation site	Family
1	HER2/ErB2	Pan-Tyr	EGFR
2	HER3/ErbB3	Pan-Tyr	EGFR
3	FGFR1	Pan-Tyr	FGFR
4	FGFR3	Pan-Tyr	FGFR
5	InsR	Pan-Tyr	Insulin R
6	Trkb/NTRK2	Pan-Tyr	NGFR
7	PDGFR	Pan-Tyr	PDGFR
8	c-Kit/SCFR	Pan-Tyr	PDGFR
9	EphA3	Pan-Tyr	EphR
10	EphB1	Pan-Tyr	EphR
11	EphB4	Pan-Tyr	EPhR
12	AKT/PKB/Rac	Thr 308	Akt
13	AKT/PKB/Rac	Ser 473	Akt
14	p44742 MAPK(ERK1/2)	Thr 202/tyr 204	MAPK
15	S6 Ribosomal protein	Ser 335/236	RSK
16	IRS-1	Pan-Tyr	IRS
17	Zap-70	Pan-Tyr	Zap-70
18	Src	Pan-Tyr	Src
19	Lck	Pan-Tyr	Src

Figure S4. Phosphorylation status of major RTKs in CHO β1 cells treated or not with ouabain. (A) The RTK Signaling Antibody Arrangement was performed with total extracts of CHO β1 cells treated with or without 50 nM ouabain for 24 h. (B) List of proteins that apparently modified their phosphorylated state in CHO β1 cells treated with 50 nM of ouabain compared to non-treated CHO β1 cells.

FIGURE S5

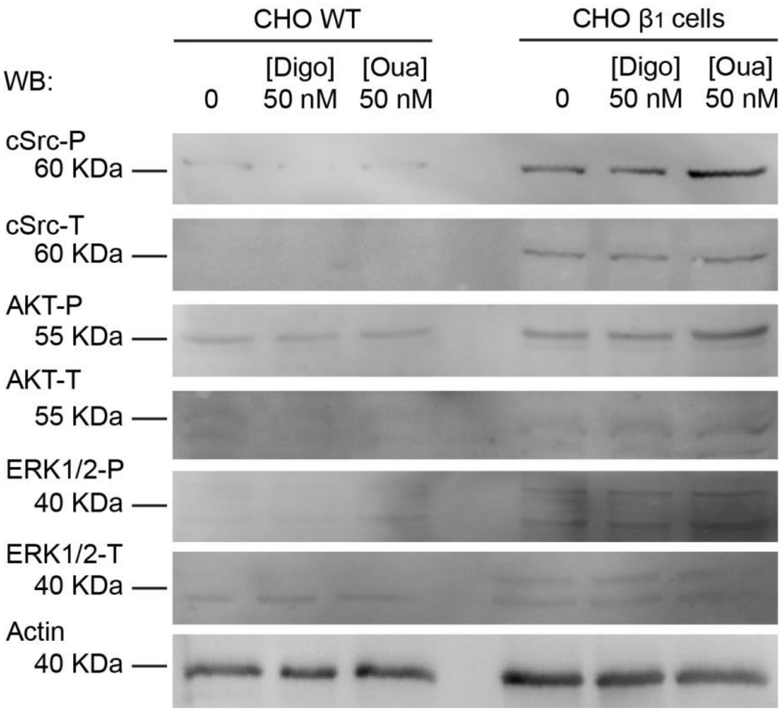

Figure S5. The effect of ouabain on activation of Src and AKT kinases in CHO β_1 cells is specific. Representative Western blot of three independent experiments of CHO WT and CHO β_1 cells treated or not with 50 nM digoxin or ouabain. The expression of total and phosphorylated cSrc, AKT and ERK1/2 kinases is depicted. Actin was used as loading control, and the blot depicted corresponds to the same shown in Figure S1, to better represent a whole set of replicates in both figures.

Supplemental methods

Phospho-RTK Antibody Array

A PathScan RTK Signaling Antibody Array Kit (7982, Cell Signaling Technology) was used according to the manufacturer's instructions to assess the phosphorylation status of the major RTKs. Briefly, cell lysate was incubated on a slide, to which was added a biotinylated detection antibody mixture, streptavidin-conjugated horseradish peroxidase, and, finally, enhanced chemiluminescent reagents. Slide images were captured with ChemiDocXRS (BioRad).
